# Declining verbal fluency after DBS in Parkinson's disease: Mental speed or executive dysfunction?

**DOI:** 10.1177/1877718X261455458

**Published:** 2026-05-30

**Authors:** Vivianne H. M. Pennings, Esmée Verwijk, Rob M. A. de Bie, Rick Schuurman, Gert J. Geurtsen

**Affiliations:** 1 Department of Medical Psychology, Neuropsychology Section, 26066 Amsterdam UMC location University of Amsterdam, Amsterdam, The Netherlands; 2 Department of Psychology, Brain and Cognition, 1234 University of Amsterdam, Amsterdam, The Netherlands; 3 Amsterdam Neuroscience, 26066 Neurodegeneration, Amsterdam, The Netherlands; 4 Department of Neurology, Amsterdam UMC location University of Amsterdam, Amsterdam, The Netherlands; 5 Department of Neurosurgery, Amsterdam UMC location University of Amsterdam, Amsterdam, The Netherlands

**Keywords:** Parkinson's disease, verbal fluency, deep brain stimulation, neuropsychological assessment, cognition, cognitive aspects

## Abstract

**Background:**

Despite the frequent decline in verbal fluency after STN-DBS surgery, the cause of decline remains unclear.

**Objective:**

This study investigates whether mental speed and executive dysfunctions are underlying mechanisms of the phonemic verbal fluency decline after STN-DBS in Parkinson's patients.

**Methods:**

Neuropsychological assessments consisting of phonemic verbal fluency, Trail Making Test (TMT), and Stroop test were conducted before and 6 months after STN-DBS in 94 Parkinson's patients in the Galaxy Trial. A Reliable Change Index (RCI) pre- versus postoperative of verbal fluency was calculated, representing the magnitude of change. Content assessment of phonemic verbal fluency resulted in content profiles (clusters and switches).

**Results:**

A statistically significant decline in postoperative phonemic verbal fluency was found, with statistically significant declined switching, while cluster size remained stable. Postoperative, statistically significant increases in time to perform TMT-B and all Stroop subtests was found. However, phonemic verbal fluency decline was correlated to change scores of Stroop I and II only. Comparisons of declined (*N* = 23) and improved (*N* = 11) patients, according to RCI's, revealed that decliners had higher preoperative phonemic verbal fluency scores.

**Conclusions:**

Postoperative phonemic verbal fluency decline could not be explained by the proposed underlying mechanisms of mental speed and executive dysfunction. However, the significant correlation of phonemic verbal fluency decline to Stroop I and II change scores suggests other possible underlying mechanisms, like slower speech.

## Plain Language Summary

Patients with Parkinson's disease receiving neurosurgical treatment afterwards frequently have a lower score on a test that measures verbal fluency: investigating possible underlying mechanisms

Parkinson's disease is a movement disorder, and among the most rapidly growing neurological disorders. Patients struggle with symptoms such as tremor, instability and rigidity. Deep Brain Stimulation is an effective neurosurgical treatment for advanced Parkinson's disease and has favorable effects on motor symptoms. However, controversy remains regarding cognitive side-effects. The most consistently reported cognitive side-effect after surgery is a lower verbal fluency. Verbal fluency is tested by asking the patient to produce as many words as possible that start with a given letter, within 60 seconds, restricted by specific rules. This study investigates which underlying mechanisms might be involved in the affected verbal fluency after neurosurgery. Specifically, issues with mental speed and executive functions were investigated as possible explaining mechanisms. Several cognitive tests were conducted before and 6 months after neurosurgery in 94 Parkinson's patients. For the verbal fluency test, besides the total number of words, it was also measured whether produced words might form categories or relationships to each other (e.g., words with the same two letters (snail, snow), rhymes (smart, start)). This was done to differentiate several (cognitive) aspects of the performance on the verbal fluency test. Also, patients' performance on tests measuring mental speed and executive functions was taken into account and compared to the aspects of the verbal fluency content. These were compared before and after surgery. The results showed that the decline on the verbal fluency test after surgery could not be sufficiently explained by the proposed underlying mechanisms of mental speed and executive dysfunction. However, slower speech was suggested as another possible mechanism. This study adds to further unravelling possible underlying mechanisms of the lower verbal fluency. A strength of this study is the large sample size increasing power and facilitating generalization.

## Introduction

Deep brain stimulation of the subthalamic nucleus (STN-DBS) is an effective treatment for patients with advanced Parkinson's disease and motor response fluctuations.^1–3^ However, with regards to the cognitive adverse effects, controversy remains. Meta-analyses showed that the most pronounced and consistently reported cognitive side-effect is verbal fluency (VF) decline after STN-DBS with noteworthy effect sizes (*d* = .51, *d* = −.49, Hedges’ *g* = −.56).^4–6^ The cause of this decline in VF remains unclear. Previous studies sought to explore the stimulation itself, microlesions, electrode positions, and changes in dopaminergic medication as possible explanations, although these remain inconclusive.^7^

A previous study found that the postoperative VF decline was accompanied by prolonged lexical decision latencies, while there were no lexical dysfunctions, and suggested that these findings point to a reduction of cognitive speed following STN-DBS in Parkinson's patients.^8^ Another study also implied a possible general cognitive slowdown after STN-DBS, after finding a significant correlation of phonemic VF decline with increased time needed on two mental speed tasks; i.e., the Trail Making Test (TMT) part A and the word card of the Stroop color-word test.^9^ Other studies found that the VF decline after STN-DBS was accompanied by decline in performance on executive processes, such as selective attention measured by Stroop color-word card^6,10,11^ and mental flexibility measured by TMT part B.^12^ Moreover, the phonemic VF task itself is also commonly interpreted as a test for executive control, aside from measuring verbal ability. Successful performance on the task not only requires access to the mental lexicon to retrieve words, but also executive control processes that are needed to focus on the selection of words that meet the criteria given in the instruction of the task, while avoiding repetition and rule-breaks.^13^ Thus, given the partly executive nature of the phonemic VF task itself, as well as the co-occurrence of decline in performance of other executive functioning tests after STN-DBS, it is possible that an executive dysfunction is an underlying mechanism responsible for the postoperative phonemic VF decline.

Besides studies of reduction of cognitive speed and executive dysfunctions, some previous studies analyzed the content of the phonemic VF.^14,15^ However, the relation of the content analysis of phonemic VF (consisting of clusters and switches) to the TMT or Stroop test in a sample size like the current study is relatively new. The content profile, formed by the verbal fluency scoring system, (i.e., phonemic and/or semantic subcategories (clusters) and their transitions (switches))^16^ offers additional information about underlying cognitive processes.^17^ Clusters are in succession produced words that belong to a described semantic or phonemic subcategory.^17^ A switch is the transition from one cluster to another cluster, or to no cluster at all. It is assumed switching relies on the frontal lobe and executive functions, such as strategy use in search processes, shifting and cognitive flexibility.^17^ Fewer switches are proposed to reflect an executive dysfunction.^17^ Thus, TMT and Stroop test, as well as the number of switches in the phonemic VF test could serve as a marker for executive (dys)function.

It is assumed clustering relies on processes of the temporal-lobe, like verbal memory and word storage.^17^ Left temporal lesions might be associated with smaller cluster sizes of semantically related words. This might be due to impaired access of the lexical information that is needed to form clusters.^16^ Previous reports showed decreased switching in phonemic VF after STN-DBS in Parkinson's disease patients, while cluster size remained stable.^14,15^

The beforementioned mechanisms of cognitive decline, overall reduction of cognitive speed after STN-DBS^8^ and executive dysfunctions,^7^ might be involved in the postoperative verbal fluency decline, as results of earlier studies remain inconclusive. A recent study, conducted in the same period as the current study, focused specifically on declining semantic (animal) VF in regards to uncovering underlying mechanisms in Parkinson's disease patients after STN-DBS surgery.^18^ This study investigated impairments in language besides executive functions, by using item-based characteristics. People with Parkinson's disease produced smaller clusters and shorter words than healthy controls after surgery, which they stress could also be explained by language and memory processing issues. In addition, patients with Parkinson's disease used more frequent words and earlier acquired words in animal fluency after surgery, compared to pre-surgery. Their item-based measures did not predict the total number of words in animal fluency after surgery. In summary, this study raises that different linguistic or cognitive aspects may be affected after surgery, while highlighting the complex nature of declining VF in Parkinson's disease.^18^ As semantic VF differs from phonemic VF the results may be incomparable. Therefore, the current study may also add to expand current knowledge of these complexities. To our knowledge, this study is one of the firsts to simultaneously study these mechanisms in phonemic VF as a main outcome together with the content analysis in a large study sample.

The current study aims to answer the question: What are the underlying cognitive processes involved in the phonemic VF decline after STN-DBS in Parkinson's patients? The VF total score and content profile containing all six variables of the Hopkins VF scoring system (e.g., clusters and switches) will be analyzed before and after STN-DBS, and related to changes on other mental speed and executive tests. We hypothesize that decline of phonemic VF is related to reduction of mental speed and/or executive function. The current study adds to existing literature a larger sample size with a more detailed content analysis than previous studies.

### The present study

For measuring mental speed, time scores of TMT part A, Stroop I and Stroop II were used. We also aim to conduct one combined (mental speed) variable based on these three tests, that takes the intercorrelations into account by means of a multivariate normative comparison (MNC) statistic.

For measuring executive functioning, time scores of TMT part B and Stroop III are used, as well as the switches variable within the phonemic VF test itself.

## Methods

### Patients

The participants of this study were recruited for the GALAXY study of the Academic Medical Center (AMC) in Amsterdam,^19,20^ which was an RCT comparing STN-DBS under general versus local anesthesia. The GALAXY study was approved by the Medical Ethics Committee (METC) of the AMC, was conducted according to the Declaration of Helsinki, and all patients provided written consent.^19,20^ Participants had idiopathic Parkinson's disease and response fluctuations. Surgical procedures included placing DBS electrodes bilaterally in the dorsolateral part of the subthalamic nucleus (for more detail see Holewijn et al.^20^). The age ranged from 36 to 73 years (*M* = 60.10, SD = 7.82, 72.30% male). Disease duration was 10.29 years (SD = 5.24). Participants did not have dementia. More detailed inclusion and exclusion criteria can be found elsewhere.^19,20^

### Neuropsychological assessment

Neuropsychological assessments were conducted before and approximately 6 months after STN-DBS. Assessments were conducted while the patient was ‘on’ medication and the follow-up assessment with STN-DBS on. For this study, the phonemic VF,^21^ the TMT^22^ and Stroop color-word test^23,24^ were investigated. For the Stroop test, performance was measured by noting the time it took for the patient to complete 100 stimuli of each subtask. For TMT, performance was also measured by noting time. When possible, parallel versions of tests were used to avoid test-retest effects. For example, for phonemic fluency, preoperative either the letters D-A-T, K-O-M or P-G-R were conducted, and postoperative one of the other remaining parallel versions was used, according to the Dutch version of the Controlled Oral Word Association Test (COWAT).^21^ The reliability is .80 and the reliability of the parallel tests is .78.^25^ Postoperative, a parallel version for TMT was used as well. For TMT, reliability varies between .61 and .79 for part A, and varies between .72 and .89 for part B.^26^ For TMT part A, test-retest reliability varying with age range and population is .69- .94 and TMT B .66−.86.^27^ According to a review,^27^ construct validity of TMT is considered good.^27^ For Stroop, test-retest reliability is above .75.^27^ Validity for Stroop was also considered adequate,^27^ as well as construct validity for phonemic VF.^27^ Correlations among phonemic VF are high from .85 to .94,^27^ and test-retest reliability is high varying with test-retest interval and fluency version from .70 to 74.^27^

#### Scoring procedure phonemic VF

The guidelines of the Hopkins VF system, as described in Ledoux et al.,^16^ are an adjustment of the traditional scoring system of Troyer et al.,^17^ and were used to analyze and score the VF answers, see Table 1A.^16^ The following variables were obtained: Total correct words, number of clusters, number of switches, total cluster size, mean cluster size, and percent words in clusters, as defined in Table 1B.^16^ As Troyer et al.^17^ previously described, the counting of the cluster size was done by starting the count with the second word in a cluster. Therefore, two successive words that form a cluster were given a cluster size of 1, and three successive words that form a cluster were given a cluster size of 2, and so forth.^17^ Further details of the scoring procedures of clusters and switches can be found in Table 1 and in Ledoux et al.^16^

**Table 1.** Hopkins verbal fluency scoring system.

**A.** *Cluster scoring guidelines*	
Rule	Example
1. Words with the same first two letters	Snail, snow
2. Words with the same first and last sounds, differing only by a vowel sound (regardless of spelling)	Simple, sample
3. Words that are homophones	See, sea
4. Words that rhyme	Smart, start
5. Words with semantic/associative relationships (a) Members of a superordinate category (b) Words that obviously hold strong associations in the real world (c) Antonyms and synonyms	Shirt, shoe, sneaker, socks Salt, shaker Sit, stand

**B.** Definition of variable*s*	
Variable	Definition
Total correct words	The sum of all words produced, minus repetitions and rule breaks
Number of clusters	The number of multiword strings. Every cluster contains at least two successive words
Number of switches	The number of transitions between clustered or non-clustered words (i.e., switches from associative strategy to another or to none at all)
Total cluster size	The sum of all clustered words
Mean cluster size	The total cluster size divided by the number of clusters
Percent words in clusters	The percentage of the total words produced (including errors) that are members of clusters.

*Note*: Adjusted table based on “Capturing additional information about the organization of entries in the lexicon from verbal fluency productions” by Ledoux et al., 2014, *Journal of Clinical and Experimental Neuropsychology*, *36*(2), p. 208

### Statistical analysis

After checking normal distributions, linearity and homoscedasticity, either paired samples *t*-tests or the non-parametric Wilcoxon matched-pairs signed rank tests were used to analyze change scores of pre- to postoperative differences. These changes scores were calculated by subtracting the baseline score from the follow-up score (i.e., phonemic VF test, Trail Making subtests and Stroop subtests). To analyze the associations between the change scores, Pearson's r was used if the data were normally distributed and homoscedastic, otherwise Spearman's rho test was used. To determine the magnitude of the (statistically reliable) change on phonemic VF scores, a Reliable Change Index (RCI)^28^ was calculated for each patient,^28^ and patients were divided in declined (RCI ≤ −1.645), stable or improved (RCI ≥ 1.645).

TMT-A, Stroop I and Stroop II were first investigated in separate analyses. However, since mental speed tests TMT part A, Stroop I and II intercorrelate, we also aimed to conduct one combined (mental speed) variable of the unstandardized (time) score of these three (sub)tests that takes the intercorrelations into account. Therefore, a multivariate normative comparison (MNC) statistic was used, comparing patients to a large normative database (Advanced Neuropsychological Diagnostics Infrastructure (ANDI: www.ANDI.nl)^29^ both pre- and postoperative. This gives insight in (entire profiles of) neuropsychological test scores. Also, a change score was calculated for this multivariate normative comparison statistic, subtracting the baseline MNC from the follow-up MNC. Furthermore, data analyses were extended with post-hoc explorative independent samples *t*-tests to examine if patients that significantly declined on phonemic VF differed on preoperative characteristics from patients that significantly improved on phonemic VF. After inspecting the corresponding assumptions, logistic regression was performed to explore the effects of age, disease duration, and the combination age×disease duration on the likelihood of phonemic VF decline.

Significance level was set at *p* < .05 for all analyses. Effect sizes were reported with *r*. Interpretation of these effect sizes follow the guidelines suggested by Cohen^30,31^; *r* = .10 for a small effect, *r* = .30 for a medium effect, and *r* = .50 for a large effect. Furthermore, the data set was examined for missing data and outliers. The latter were defined as values more than 3.29 SD below or above the mean.^32^ To handle these outliers, scores were first winsorized, and assigned a less significant value of ±3 SD, to limit the impact of these outliers on the results.^32^ All statistical analysis were performed with IBM SPSS 26.

## Results

### Participants

The current study existed of 99 participants, of which 94 were included. Five patients were excluded: four due to missing data, and one due to a possible distortion in neuropsychological test scores due to a mild depressive disorder after STN-DBS. The age ranged from 36 to 73 years (*M* = 60.10, SD = 7.82, 72.30% male), a disease duration of 10.29 years (SD = 5.24) and median education level of 5 on the Verhage scale.^33^

### Cognitive performance

The mean duration between STN-DBS and the follow-up neuropsychological assessment was 6.59 ± 1.29 months.

After further inspection of the cognitive variables, seven patients were detected to be outliers, with values above or below 3.29 SD on one or two variables. Furthermore, histograms, boxplots, PP-plots, and descriptive values of skewness and kurtosis showed that change scores of TMT part A, Stroop I, phonemic VF total correct score, number of clusters, number of switches, cluster size, and mean cluster size were approximately normal distributed. Change scores of Stroop II, Stroop III, and TMT part B were not normal distributed. Scatterplots showed that the distributions were approximately linear. Not all were homoscedastic.

The following results all compare post STN-DBS scores to baseline scores. There was a statistically significant increase in the amount of time needed to complete all Stroop subtests. Effect sizes of Stroop I and II were large (*r* = .45 and *r* = −.48 respectively), and of Stroop III was medium (*r* = −.39). After STN-DBS, there was no significant difference in the amount of time needed to complete TMT part A. There was a statistically significant increase in the postoperative amount of time needed to complete TMT part B. The effect size was small (*r* = −.18). The postoperative MNC (combination Stroop I, II, & TMT part A) increased statistically significantly compared to the preoperative MNC. Neuropsychological test scores and multivariate normative comparison (MNC) can be found in Table 2.

**Table 2.** Pre- and postoperative neuropsychological test scores.

	Preoperative score	Postoperative score	*p* value
Phonemic verbal fluency	38.38 ± 11.95	36.12 ± 12.28	**.007** ^a **,** c^ *
- Number of clusters	6.96 ± 3.85	6.82 ± 3.22	.678^d^
- Number of switches	29.71 ± 9.90	26.59 ± 10.65	**<.001** ^c^ *
- Total cluster size	9.49 ± 6.12	9.98 ± 5.42	.380^d^
- Mean cluster size	1.35 ± .36	1.42 ± .49	.185^d^
- Percent words in clusters	38.04 ± 17.06	41.73 ± 15.12	.062^d^
Stroop I	45.39 ± 8.75	48.87 ± 10.62	**<.001** ^a **,** d^ *
Stroop II	60.98 ± 10.95	69.16 ± 16.15	**<.001** ^b **,** d^ *
Stroop III	105.07 ± 30.27	129.90 ± 68.26	**<.001** ^b **,** c^ *
TMT part A	37.20 ± 16.04	37.66 ± 20.21	.385^a,c^
TMT part B	109.12 ± 76.42	127.12 ± 114.19	**.006** ^b**,**c^ *
MNC	1.14 ± .92	1.85 ± 1.78	**.003** ^b**,**d^ *

*Note:* Values are presented as mean ± standard deviation (SD). Abbreviations: TMT, Trail Making Test, MNC, Multivariate Normative Comparison

^a^ Paired-samples *t*-test

^b^ Wilcoxon matched-pairs signed rank test

^c^ One-tailed value

^d^ Two-tailed value

*Significant at *p* < .05

There was a statistically significant postoperative decline of phonemic VF scores (Table 2). The effect size was medium (*r* = .25).

Based on the RCI, twenty-three patients (24.47%) showed a significant decline and 11 (11.70%) showed a significant improvement in phonemic VF. The significantly declined group and the significantly improved group did not differ on preoperative characteristics such as demographic characteristics, and preoperative Stroop and TMT subtest scores (Table 3). However, the declined group had statistically significantly higher preoperative phonemic VF scores than the improved group. The effect size was medium (*r* = .38). There was a statistically significant decline in the number of switches with a medium effect size (*r* = .33). There were no significant differences in the total cluster size, mean cluster size, number of clusters, nor percent words in clusters.

**Table 3.** Baseline characteristics of three groups of phonemic verbal fluency.

	Declining group RCI ≤ −1.645 (*N* = 23)	Improving group RCI ≥ 1.645 (*N* = 11)	*p* value	Remaining group RCI −1.645↔1.645 (*N* = 60)
Age (years)	57.83 ± 7.60	53.45 ± 9.05	.15	62.18 ± 6.80
Education level (Verhage)	5 (median)	5 (median)	.83	6 (median)
Disease duration (years)	10.83 ± 6.41	7.91 ± 2.84	.16	10.52 ± 5.04
Phonemic verbal fluency	44.13 ± 12.81	33.64 ± 10.78	.03*	37.05 ± 11.20
Stroop I	44.61 ± 9.31	41.45 ± 8.10	.34	46.42 ± 8.55
Stroop II	60.52 ± 12.80	58.73 ± 11.81	.70	61.57 ± 10.15
Stroop III	97.91 ± 28.21	96.00 ± 23.77	.85	109.48 ± 31.56
TMT part A	38.74 ± 18.56	31.00 ± 12.33	.22	37.75 ± 15.58
TMT part B	109.43 ± 64.88	81.00 ± 38.50	.12	114.15 ± 84.93

*Note:* Values are presented as mean ± standard deviation (SD). Abbreviations: TMT, Trail Making Test. RCI, Reliable Change Index.^24^ Education level is scored on a scale ranging from 1 (low) to 7 (high).^29^

*Significant at *p* < .05

### Exploring underlying mechanisms

A significant relationship between the change score of phonemic VF and the change score of Stroop I and II respectively was found (Table 4). However, when the analysis was run again without the outlier on Stroop II, this was no longer significant, *r* = −.11, *p* (one-tailed) = .052. The change score of phonemic VF was not significantly related to the change score of Stroop III, nor to change scores of TMT part A and part B, nor the change score of the MNC. Lastly, the logistic regression model to explore if age, disease duration and the combination age X disease duration could predict phonemic VF decline was not significant.

**Table 4.** Correlations of phonemic verbal fluency and Stroop + TMT change scores.

	Change score phonemic verbal fluency	
	Correlation coefficient^a,b^	*p* value (one-tailed)
Change score Stroop I	−.18^a^	.046*
Change score Stroop II	−.19^b^	.034*
Change score Stroop III	−.14^b^	.084
Change score TMT part A	.05^a^	.318
Change score TMT part B	−.05^b^	.334
Change score MNC	.03^b^	.374

*Note*: Change score = follow-up score – baseline score. Hence, negative phonemic verbal fluency change scores indicate a decline in performance and positive Stroop and TMT change scores (increased in time) indicate a decline in performance. Abbreviation: TMT, Trail Making Test, MNC, Multivariate Normative Comparison

^a^ Pearson's *r*

^b^ Spearman's rho

*Significant at *p* < .05

## Discussion

In this study we investigated the cognitive processes involved in the phonemic VF decline after STN-DBS in Parkinson's patients. We proposed mental speed and executive dysfunctions as underlying mechanisms. In line with previous literature, we found a significant postoperative phonemic VF decline.^4–6^ Of the 94 included patients 24.5% showed a significant decline and 11.7% showed a significant improvement.

There was a significant decline in the number of switches. Thus, there were less postoperative transitions from one cluster to another cluster, or to no cluster at all. Since switching relies on executive functions,^17^ this seems to be affected. However, regarding executive tests, no significant relation was found of change scores of TMT part B and Stroop III and the phonemic VF decline. Furthermore, there were no significant differences in the total cluster size, mean cluster size, number of clusters, nor percent words in clusters. Thus, the access of lexical information that is needed to form clusters seems unaffected.^16^ No sufficient support for all of our hypotheses of mechanisms of mental speed and executive dysfunction was found.

### Mental speed mechanism

The combined mental speed tests (Stroop I, II, & TMT part A) showed a significant increase in the multivariate statistic after STN-DBS. This was driven by the significant increase in the amount of time needed to complete both Stroop subtests, but not by TMT part A. As the Stroop tests have a mental speed and speech component, for mental speed to be the underlying cognitive mechanism, an increased amount of time to complete TMT part A was also considered to be required. However, there was no significant difference in the amount of time needed to complete TMT part A. Nevertheless, one might argue that this lack of difference might be explained by the improved motor functions, that can compensate for possible postoperative mental speed dysfunctions, thus preventing decreased performance on the TMT part A. Furthermore, the change score in TMT part A was not significantly correlated with the postoperative decline in phonemic VF. This was not in line with a small previous study that did find a significant correlation of decreased phonemic VF with increased time needed on TMT part A.^8^

The postoperative significant increase in the amount of time on Stroop I and II is in line with previous studies.^9,10,34^ Stroop I and II change scores were significantly correlated to the phonemic VF decline. It should be noted that the correlation of Stroop II change score to phonemic VF decline was no longer significant when the analysis was run again without the (corrected value) outlier on Stroop II, and results must therefore be interpreted with caution.

Thus, for a mental speed dysfunction, not all required hypotheses were supported: there was no support by TMT part A, but results did support Stroop I and II hypotheses. Since speech functions are needed in the Stroop subtests, and speech can be impaired after STN-DBS,^35^ this might be an alternative underlying mechanism.

### Executive dysfunction mechanism

For the executive mechanism to play a role in the VF decline, this needs to be substantiated by 1. the relation of phonemic VF to executive tests (i.e., the scores on TMT part B and Stroop III), and 2. a decline in number of switches. There was indeed a significant increase in time needed to complete TMT part B and Stroop III, after STN-DBS. However, change scores of TMT part B and Stroop III were both not significantly correlated to the phonemic VF decline. However, there was a significant postoperative decline in switches, and no significant change in cluster size. This finding was in line with previous studies.^14,15^ Thus, after STN-DBS the ability to switch between phonemic or semantic subcategories, or to noncategorized words was affected. This is assumed to reflect an executive dysfunction.^17^ Since only one of the two executive mechanisms was supported, one might speculate that the executive functions differ in nature. Executive functions like response inhibition in Stroop III, and divided attention in TMT part B^26^ might differ from the strategy use in search processes, shifting and cognitive flexibility needed in switching in VF tasks.^17^ Using a more precise defined executive dysfunction in combination with functional and structural analyses^36,37^ might be more appropriate in future studies to explain the underlying mechanism of the postoperative phonemic VF decline.

When analyzing the content profile more in detail, it becomes apparent that a trend can be discovered of a postoperative higher percent words in clusters. However, because this trend is not significant, no solid conclusions can be drawn and it must be interpreted with caution. When future research, in for example a larger study sample, would confirm this higher percentage and a decline in switches, it would become more likely that executive dysfunction might still play a role as an underlying mechanism of (language) inflexibility STN-DBS.

### Other possible mechanisms and predictors

Post-hoc explorations revealed that the patients that significantly declined on phonemic VF differed on preoperative characteristics from the patients that significantly improved. The declining group had significantly higher preoperative phonemic VF scores. Some trends were seen when comparing both groups. However, these trends are not significant and no solid conclusions can therefore be drawn. The declining group tended to be older in age, and seemed to have a longer disease duration than the improving group. A previous study also showed that the risk of VF declines after STN-DBS increased with higher age.^38^ In addition, the declining group tended to have a higher score on TMT part B, which represented a worse performance. These tendencies should be interpreted with caution, since these subgroups consisted of small sample sizes. Future studies could further investigate possible demographic (e.g., age, education level) and clinical (e.g., neuropsychological tests) preoperative differences between these groups in larger sample sizes, and thus possibly detect predictors of the postoperative VF decline.

### Final recommendations

Future studies could further unravel the possible relation of speech pattern changes and phonemic VF word generation. Previous studies proposed that word generation during phonemic fluency can be divided in two distinct processes. Word production in the initial stage relies on a semiautomatic retrieval process, and is more efficient. Other quartiles involve processes more effortful, and slower, while the patient is adjusting, monitoring and planning the generated words and avoiding intrusions or repetitions.^39–41^ Overall, word generation descends over the 60 s period. A previous study^42^ found that fewer words were produced in the initial stage by individuals with a Mild Cognitive Impairment, compared to a healthy control group, and implied the decline in this less demanding stage might be attributed to a slowing of the information processing rate and retrieval of mental lexicon. A study^43^ investigating Parkinson's disease patients, showed a tendency for a slight decline, though not significant, in phonemic fluency word production during the initial stage after STN-DBS, compared to preoperative results. Future studies could investigate possible changes in speech patterns and pace before and after STN-DBS, and relate these to the postoperative phonemic VF decline, as speech functions can be impaired after STN-DBS surgery.^35^

This may be combined with a localization study. As an earlier study found DBS in the medial and posterior regions of the STN to influence VF, while DBS in the posterior and lateral regions of the STN influenced motor function.^44^ The authors claimed that given the current DBS electrode size, all these regions are concurrently activated. Another localization study found that postoperative verbal fluency decline was associated with the position of the left active electrode along the dorsal-ventral axis in STN. The patients with the largest declines in VF were stimulated in a relatively more dorsal subregion of the STN.^45^ Hence, future directional and targeted stimulation might offer a solution for Parkinson's patients to benefit from motor effects, while avoiding the side-effect of postoperative VF declines.^44,45^

### Strengths and limitations

A strength of this study is the large sample size that increases the power of the analyses, and facilitates generalization of the found effects. Also, practice effects were reduced by the use of parallel versions of the tests. The practice effects that remained (e.g., being familiar with the task despite the use of parallel versions) strengthen the robustness of the results, since postoperative declines in phonemic VF performance and increased amount of time in TMT and Stroop were found, despite practice effects. Therefore, it can be expressed with more confidence that the found results were due to systematic variance after STN-DBS.

A limitation of the current study is that assessment of the cognitive domains mental speed and executive functions could solely be associated to Stroop and TMT performances. A more comprehensive battery of neuropsychological tests, assessing components of mental speed and executive functioning in a more granular way and considering factor analysis, while also including other measures of cognitive flexibility, inhibition, working memory, as well as vocabulary and naming tests is recommended for future studies as well as measurement of speed of pronunciation, for a better characterization of the cognitive profile of patients and a deeper understanding of the underlying mechanisms of changes in verbal fluency. In addition, since only phonemic verbal fluency was assessed, future research might also consider adding semantic VF tasks. Another limitation was that the current study did not have a control group.

## Conclusion

In conclusion, no sufficient support was found to explain the postoperative phonemic VF decline with the proposed mechanisms of mental speed and executive dysfunction. The speed of pronunciation of the words in phonemic VF might play a role, and can be investigated in future research.
